# Dynamics of Sun5 Localization during Spermatogenesis in Wild Type and *Dpy19l2* Knock-Out Mice Indicates That Sun5 Is Not Involved in Acrosome Attachment to the Nuclear Envelope

**DOI:** 10.1371/journal.pone.0118698

**Published:** 2015-03-16

**Authors:** Sandra Yassine, Jessica Escoffier, Roland Abi Nahed, Virginie Pierre, Thomas Karaouzene, Pierre F. Ray, Christophe Arnoult

**Affiliations:** 1 Université Grenoble Alpes, Grenoble, F-38000, France; 2 Equipe "Génétique, Epigénétique et thérapies de l’Infertilité" Institut Albert Bonniot, INSERM U823, Grenoble, F-38000, France; 3 CHU de Grenoble, UF de Biochimie et Génétique Moléculaire, Grenoble, F-38000, France; Nanjing Medical University, CHINA

## Abstract

The acrosome is an organelle that is central to sperm physiology and a defective acrosome biogenesis leads to globozoospermia, a severe male infertility. The identification of the actors involved in acrosome biogenesis is therefore particularly important to decipher the molecular pathogeny of globozoospermia. We recently showed that a defect in the *DPY19L2* gene is present in more than 70% of globozoospermic men and demonstrated that Dpy19l2, located in the inner nuclear membrane, is the first protein involved in the attachment of the acrosome to the nuclear envelope (NE). SUN proteins serve to link the nuclear envelope to the cytoskeleton and are therefore good candidates to participate in acrosome-nucleus attachment, potentially by interacting with DPY19L2. In order to characterize new actors of acrosomal attachment, we focused on Sun5 (also called Spag4l), which is highly expressed in male germ cells, and investigated its localization during spermatogenesis. Using immunohistochemistry and Western blot experiments in mice, we showed that Sun5 transits through different cellular compartments during meiosis. In pachytene spermatocytes, it is located in a membranous compartment different to the reticulum. In round spermatids, it progresses to the Golgi and the NE before to be located to the tail/head junction in epididymal sperm. Interestingly, we demonstrate that Sun5 is not, as initially reported, facing the acrosome but is in fact excluded from this zone. Moreover, we show that in Dpy19l2 KO spermatids, upon the detachment of the acrosome, Sun5 relocalizes to the totality of the NE suggesting that the acrosome attachment excludes Sun5 from the NE facing the acrosome. Finally, Western-blot experiments demonstrate that Sun5 is glycosylated. Overall, our work, associated with other publications, strongly suggests that the attachment of the acrosome to the nucleus does not likely depend on the formation of SUN complexes.

## Introduction

The acrosome, is a specialized organelle allowing the sperm to cross the zona pellucida. Acrosome biogenesis is a complex event which begins with the formation of the acroplaxome, a specific cytoskeletal structure composed of a network of proteins which includes keratin 5 and beta-actin [1]. The acroplaxome binds to the nuclear envelope while specific vesicles produced by the Golgi apparatus are targeted to the acroplaxome where they fuse to generate the acrosomal vesicle. Additional vesicles then fuse with the growing acrosomal vesicle allowing its stretching during spermatid elongation. The mechanism permitting the association of the different components of the acroplaxome and their attachment to the nuclear envelope on one side and to the acrosome on the other side remain largely uncharacterized [2].

Globozoospermia is a rare genetic disorder characterized by the absence of the acrosome in sperm head. It constitutes an interesting model to study acrosome biogenesis and to identify the different molecular actors involved in sperm head formation. We recently showed that the deletion or mutations of *DPY19L2* are responsible for 70% of human type I globozoospermia [3,4] and that the corresponding protein plays a crucial role in stabilizing the attachment of the acrosome during acrosome stretching associated with spermatid elongation [5]. This result strongly suggests that other proteins are involved in the initial binding of acroplaxome on the nuclear envelope. LINC proteins (LInkers of the Nucleoskeleton to the Cytoskeleton), composed of Sun proteins on the inner nuclear membrane (INM) and Kash on the outer nuclear membrane (ONM), are particularly interesting candidates since their function is to position the nucleus within the cell via cytoskeletal interactions but also to tether centrosome and chromosomes to the nuclear envelope [6,7]. The LINC proteins located in the INM are called Sun proteins (Sad1/UNc-84 homology) and those located in the ONM, Kash proteins (Klarsicht, Anc-1, and Syne/nesprin Homology). In Mammals, there are five different Sun proteins, Sun1, Sun2, Sun3 (also called SunC1), Sun4 (also called Spag4) and Sun5 (also called Spag4l). The N-terminus of the Sun proteins is located in the nucleoplasm and interacts with different proteins of the nucleoskeleton; the C-terminus, located in the lumen of the NE, interacts with C-terminus of the Kash protein and the N-terminus of the Kash protein interacts with different proteins of the cytoskeleton. The formation of theses complexes thus allows a direct mechanical coupling between proteins located inside the nucleus, such as lamins A,C (but not with lamin B) to proteins located in the cytoplasm such as actin, plectin or dyneins.

Sun1 and Sun2 are ubiquitously expressed [7]. Sun4 shows a low expression level in many tissues except in the lymph node and in the testis where it is expressed at a higher level [8]. Sun3 and Sun5 expression is restricted to the testis. All five Sun proteins are thus expressed in the testis. Interestingly, the Sun proteins present a remarkable differential localization in the spermatid. During spermiogenesis, Sun1 localization is restricted to the posterior part of the spermatid where it interacts with Syne3 Kash protein. Sun1 is however excluded from the implantation fossa, the articulation which serves to attach the neck (and the rest of the flagella) to the head. Sun3 is absent from the posterior and anterior poles of the elongating spermatid and colocalizes with the microtubules of the manchette along the lateral NE and interacts with the Syne1 Kash protein [9]. Concerning Sun4, the protein is located in the manchette area during spermiogenesis and in the flagellum in mature sperm cell [8]. Its function in NE is however not described in mammals so far. Interestingly, the ortholog of Sun4 in *Drosophilia* is required for correct positioning of the centriole during spermatogenesis. This result strongly suggests a specific role of Sun4 protein during spermatogenesis [10]. Finally, Sun5 is expressed during spermiogenesis. It is described to present five alternative transcripts but their differential expression pattern has not been investigated. Contradictory results were published regarding Sun5 expression and localization. A first report has shown that Spag4l (alias of Sun5) and Spag4l-2 (a longer isoform of the same gene) are expressed after the meiosis and are located in the apical nuclear envelope of round spermatids facing the acrosome [11] whereas a second study reported an early and strong expression during meiosis [12]. Sun5 could therefore be one of the missing link between the nuclear envelope and the acroplaxome and may, with Dpy19l2, participate to the anchoring of the acroplaxome to the NE. The characterization of these missing links is particularly important in the context of type I globozoospermia, since around 30% of cases are still unexplained. To address this question, we re-evaluated the localization of Sun5 during spermatogenesis. In this paper, we first demonstrate that Sun5 is present at the pachytene stage within the cytoplasm and migrates to the NE at the round spermatid stage. We demonstrate however that, contrary to previously published work [11], Sun5 is not located in the NE facing the acrosome. Moreover, we show that in Dpy19l2 knock-out round spermatids, the detachment of the acrosome leads to Sun5 and lamin B1 relocalization, confirming that Sun5 protein is excluded from the NE facing the acrosome. We therefore show that Sun5 cannot be a functional partner of Dpy19l2, strongly suggesting that Sun5 is not involved in acrosome attachment. In round and elongated spermatids, Sun5 presents a localization that is partly similar to Sun1 and 3. In epididymal sperm, it moves to the junction area between the tail and the sperm head. Finally, we show that Sun5 transits through the Golgi apparatus, leading to post-translational modifications, as witnessed by the appearance of a glycosidase-sensitive band in Western-blot experiments.

## Results

**Fig 1:**
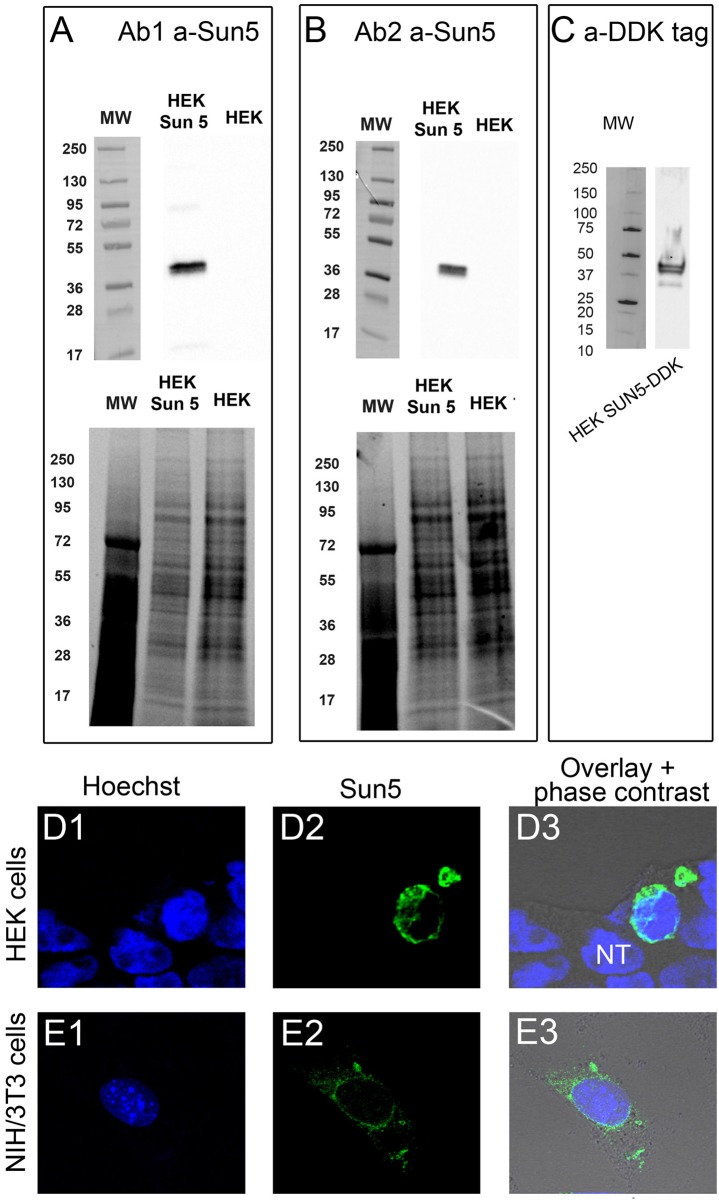
Specificity of antibodies targeting Sun5. **(A)** Western blot of protein extracts from HEK cells heterologously expressed with or without plasmid containing mouse short isoform of Sun5 and revealed with anti-Sun5 antibodies 1 (Ab1). Below, gel of protein showing that both lanes were similarly loaded. **(B)** Similar experiment but revealed with anti-Sun5 antibodies 2 (Ab2). **(C)** Western blot of protein extracts from HEK cells heterologously expressed with a plasmid containing human short isoform of SUN5-DDK tagged and revealed with anti-DDK antibodies. **(D)** HEK cells were transfected with mouse short isoform of Sun5 and stained with Hoechst (blue, D1) and anti-Sun5 Ab1 (D2, green). D3 corresponds to overlay + phase contrast. **(E)** Similar experiment performed with NIH/3T3 cells.

**Fig 2:**
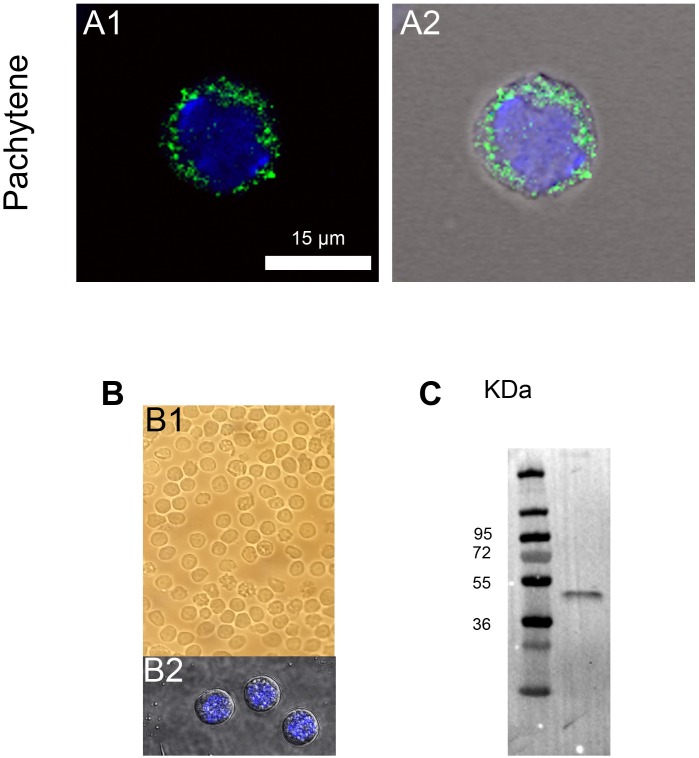
Sun5 is located in the cytoplasm in pachytene spermatocyte. (**A**) Pachytene spermatocyte co-stained with Hoechst (blue) and anti-Sun5 Ab1 (green) (A1). A2 corresponds to overlay + phase contrast. **(B)** Pachytene spermatocytes were purified by unit gravity sedimentation from spermatogenic cell suspension obtained from sexually mature males. The purity of the fraction (B1, observed with optical microscope), was assessed by counting the cells exhibiting characteristic Hoechst nuclear staining under a fluorescent microscope (B2). **(C)** Western blot of cytoplasmic proteins extracted from the pachytene spermatocytes showing that Anti-Sun5 Ab immunodecorates a single band around 40 KDa.

In order to study the potential role of Sun5 during spermatogenesis, we first characterized two different antibodies targeting different epitopes, one from a commercial company (Ab1) and one laboratory designed (Ab2). HEK cells were first transfected with the Sun5 plasmid (mouse Spa4l, short isoform). Gels of proteins, revealed separately with both antibodies, presented the same profile of bands in Western blotting experiments: two close bands were immunodecorated, with an approximate molecular weight of 40 KDa (Fig. 1AB). These two bands are also clearly evidenced with protein extracts from HEK cells, transfected with a plasmid containing the human SUN5, tagged with DDK tag and revealed with anti-DDK antibody (Fig. 1C). DDK is a low molecular weight tag (DYKDDDDK (1012 Da), known to not interfere with protein function (expected MW of Sun5-DDK is 40.5 KDa). In contrast, blots from non transfected HEK cells showed no bands (not shown), demonstrating the high specificity of these three antibodies. Both Ab1 and Ab2 antibodies were then tested in IHC experiments and signals were observed by confocal microscopy. Ab1 marked strongly the nuclear envelope in both HEK and NIH/3T3 cells transfected with Sun5 plasmid, as expected for a protein belonging to the Sun family (Fig. 1DE). We observed in NIH/3T3 cells a fluorescent signal around the nucleus but also in the cytoplasm (Fig. 1E2-E3). This latter result suggests that Sun5 may be located in the reticulum or traffic through other membrane compartments (Golgi apparatus, ERGIC, …). Remarkably, the non transfected cells (NT) presented no fluorescence, either around the nucleus or in the cytoplasm (Fig. 1D3). We also performed IHC control experiments on non transfected HEK cells, and similarly no staining was observed (not shown). Finally, Ab2 didn’t work in IHC. Altogether, these results demonstrate that our antibodies present a high specificity in WB and that Ab1 is a valuable tool to study Sun5 protein localization in spermatogenic cells by IHC. Then, we studied the expression and localization of Sun5 in different dissociated spermatogenic cells, from the pachytene stage to mature sperm cells. We first analyzed the presence of Sun5 in pachytene spermatocyte by IHC experiments: at this stage a punctiform staining was observed only within the cytoplasm (Fig. 2A, see also S2 Fig.). It is worth noting that no staining was observed in control experiments performed on different spermatogenic cells with only the secondary Ab (S1 Fig.). This staining is coherent with that obtained in NIH/3T3 cells and strongly suggests that Sun5 is not located in the nuclear envelope but rather within the cytoplasm at this stage. In order to validate the cytoplasmic Sun5 staining, cytoplasmic proteins extracted from dounce homogenized pachytene spermatocytes were subjected to SDS page and revealed with Sun5 Ab2 (n = 4). Protein extracts were prepared from pachytene spermatocytes purified by unit gravity sedimentation from spermatogenic cell suspension and obtained from sexually mature males. The purity of the fraction was assessed by counting the cells exhibiting characteristic Hoechst nuclear staining under a fluorescent microscope (Fig. 2B) and we estimated that 95% of the cells were indeed pachytene spermatocytes. Ab2 immunodecorated a single band at 40 KDa, the expected MW of Sun5 (Fig. 2C). To better characterize the localization of Sun5 within the cytoplasm, a co-localization experiment of Sun5 with a reticulum marker (KDEL antibodies) was performed. Clearly, most of the Sun5 staining did not co-localize with the KDEL staining at the pachytene stage (S2 Fig.), indicating that Sun5 is located in another membranous compartment.

**Fig 3:**
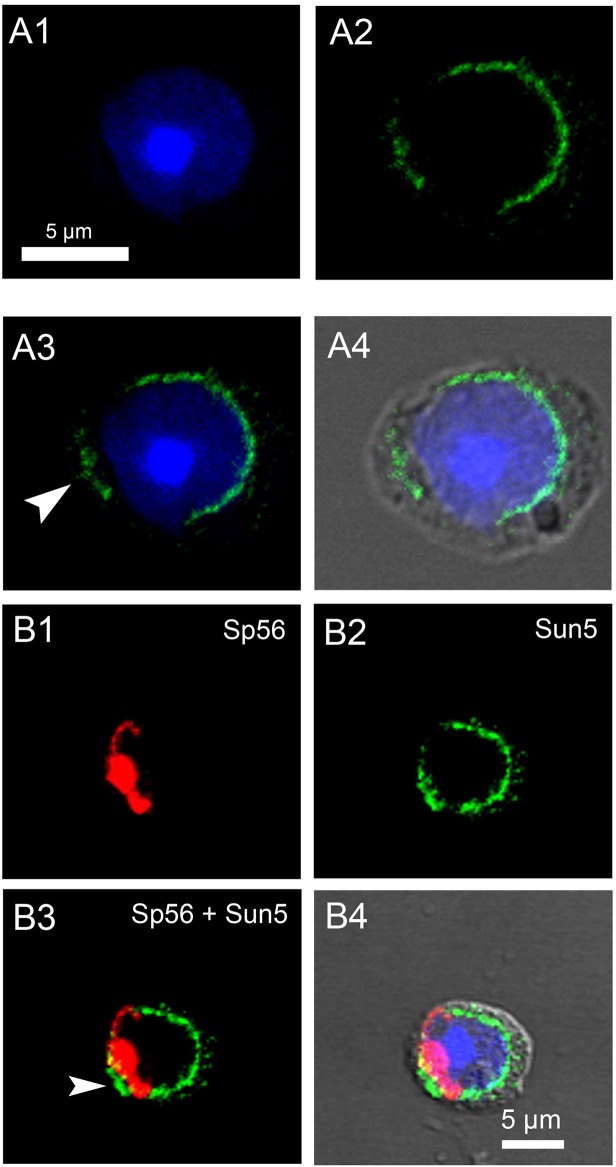
Sun5 is located in the nuclear envelope (NE) in round spermatid and is excluded from the NE facing the acrosome. (**A)** Round spermatid stained with anti-Sun5 Ab1 (A2, green) and counterstained with Hoechst to evidence the nucleus (A1, blue). A3 corresponds to overlay of A1 and A2; Arrow head indicates the Sun5 staining which was not bound to the NE. A4 corresponds to overlay with phase contrast. **(B**) Round spermatid co-stained with anti-Sp56 (B1, red) and anti-Sun5 Ab1 (B2, green) and counterstained with Hoechst to evidence the nucleus (blue). B3 corresponds to overlay of B1 and B2; arrow head indicates the Sun5 staining which was not bound to the NE. Note that Sun5 staining is located in a more external location than Sp56 staining. B4 corresponds to overlay with phase contrast.

**Fig 4:**
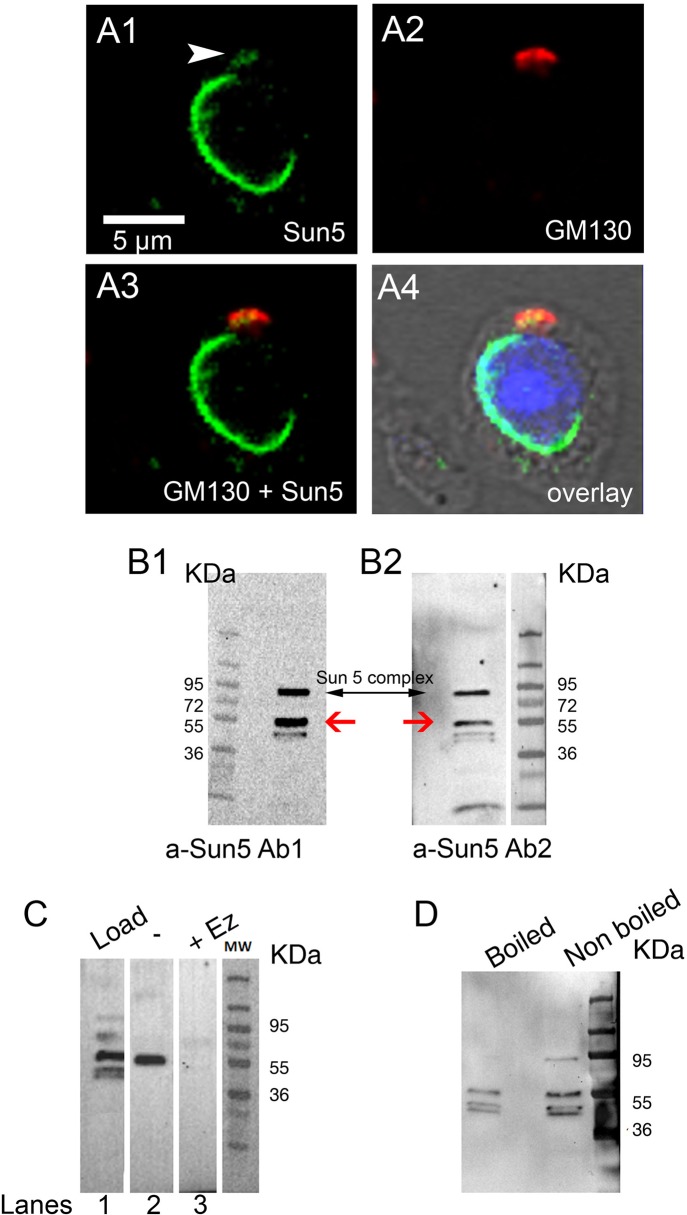
Sun 5 progresses through the Golgi apparatus and presents post-translational modifications during spermiogenesis. **(A)** Round spermatid co-stained with anti-Sun5 Ab1 (A1, green) and anti-GM130 antibodies a marker of the Cis-Golgi (A2, red) and counterstained with Hoechst to evidence the nucleus (blue). A3 corresponds to overlay. A4 corresponds to overlay with phase contrast **(B)** Western blots of nuclear protein extracts from testis revealed with anti-Sun5 Ab1 (B1, left) and anti-Sun5 Ab2 (B2, right), showing a similar pattern evidenced by both antibodies. **(C)** Protein extracts (load, lane 1) were incubated 15 hours without (−, lane 2) or with a deglycosylation enzyme mix (+ Ez, lane 3) and the impact on Sun5 was studied in Western blot with Ab2 antibodies. **(D)** Protein extract was split in two identical fractions, and one fraction was boiled. The band at 95 KDa observed with Sun5 antibodies in Fig. 4B was no longer present when denaturation conditions were strengthened by boiling the sample in Laemmli buffer. Protein loads controlled with TGX stain free precast gels are showed in panel C of S5 Fig.

The progression of meiosis to the round spermatid stage led to an important relocalization of Sun5 from the cytoplasm to the nuclear envelope (Fig. 3A), as witnessed by Sun5 staining which exhibited an incomplete ring shape at the nuclear periphery (Fig. 3A). More than 90% of round spermatids presented a discontinuous staining on the NE. In order to better characterize this incomplete ring, we performed a co-staining with anti-Sp56 antibodies, a marker of the acrosomal vesicle [13]. In fact, most of the NE facing the acrosome was not marked by anti-Sun5 antibodies (Fig. 3B, see also panels A-D of S3 Fig.), and acrosome binding clearly seems to exclude Sun5 from the NE facing the acrosome. Moreover, part of the signal was not attached to the nuclear membrane and was located at a short distance from it (see head arrows, Fig. 3A3), an unexpected observation for a Sun proteins normally located in the INM [14]. In overlay with phase contrast, this signal was clearly located in the cytoplasm (Fig. 3A4). When cells were co-stained with Sun5 and Sp56 Abs, this peculiar Sun5 staining, unbound to the nuclear envelope, was always located in the vicinity of the acrosome (Fig. 3B3, arrow head). Additional examples of the presence of NE-unbound Sun5 staining are showed in panels A3,B3,E3 of S3 Fig. (white and yellow arrow heads). Superimposition of Sun5 and Sp56 NE-unbound staining evidenced that Sun5 staining was always located more externally than the Sp56 acrosomal staining (Fig. 3B3, white arrow head, see also panel E of S3 Fig., yellow arrow head). This remark is important because we have previously shown that the staining of Dpy19l2, a protein of the nuclear membrane involved in acrosome anchoring, was always more internally located than the staining of the acrosome in experiments performed similarly [5]. The fact that Sun5 staining is located more externally than Sp56 staining suggests that this peculiar Sun5 staining did not correspond to the NE facing the acrosome nor to the acrosome itself. This localization is compatible with the Golgi apparatus, which is located in front of the acrosome. We tested this hypothesis by performing co-staining experiments with Anti-Sun5 and anti-GM130 Abs, a marker of the Cis-Golgi. We observed a strong co-localization of GM130 and the NE-unbound Sun5 staining, demonstrating that Sun5 is targeted to the Cis-Golgi (Fig. 4A1–4). Other examples of representative co-localization of NE-unbound Sun5 staining and GM130 in round spermatids are presented in S4 Fig. This co-localization was observed in almost all round spermatids presenting a NE-unbound Sun5 staining. In the Golgi apparatus, numerous proteins are modified by the addition of carbohydrates (glycosylation) or phosphates (phosphorylation). The presence of Sun5 in the Cis-Golgi therefore suggests that Sun5 could be subjected to such post-translational modifications. This is in agreement with the presence of the two close bands observed when the Sun5 plasmid was transfected in HEK cells (Fig. 1AB). To address a possible glycosylation of Sun5 during its transit through the Golgi apparatus, nuclear protein extracts from spermatogenic cells were subjected to SDS page followed by Western blot analysis. Interestingly, in these extracts, we confirmed the presence of a doublet around 40 kDa but we also evidenced a new band around 55 kDa, immunodecorated by both antibodies (Fig. 4B, red arrows). The fact, that both antibodies immunodecorated the same band, demonstrates that the signal is likely specific and shows that Sun5 is strongly modified during its transit through the Golgi apparatus. Numerous N and O glycosylation sites are predicted to be present on the protein and the band at 55 KDa could correspond to a glycosylated protein. To address this question, we incubated testis extracts during 15 hours at 37°C with a mix of deglycosylation enzymes. First we controlled that our protocol was able to remove glycosyl residues by using bovine fetuin as a glycosylated control protein (panel A of S5 Fig.). Contrary to testis extracts incubated without enzymes (Fig. 4C, lane 2), the band at 55 KDa was no longer observable after deglycosylation treatment (Fig. 4C, Lanes 3), confirming that the band at 55 KDa corresponds to a post-translationnaly modified Sun5. Unexpectedly, the process of incubation by itself (i.e. without enzymes), modified the Sun5 signal: the doublet at 40 KDa was no longer observable (Fig. 4C, Lanes 1 versus 2), suggesting that Sun5 is highly sensitive to degradation when it is deglycosylated. Protein loads before (lane 1) and after treatment (lanes 2 and 3) were controlled with TGX stain free precast gels and no dramatic differences were observed (Panel B of S5 Fig.). Moreover, a band around 95 KDa was also observed (Fig. 4B, black arrow) which likely corresponds to a protein complex containing Sun5 because this band was no longer present when the reducing conditions were increased by boiling the sample before gel loading (Fig. 4D). Protein loads, controlled with TGX stain free precast gels, shows that similar amount of protein were loaded (panel C of S5 Fig.).

**Fig 5:**
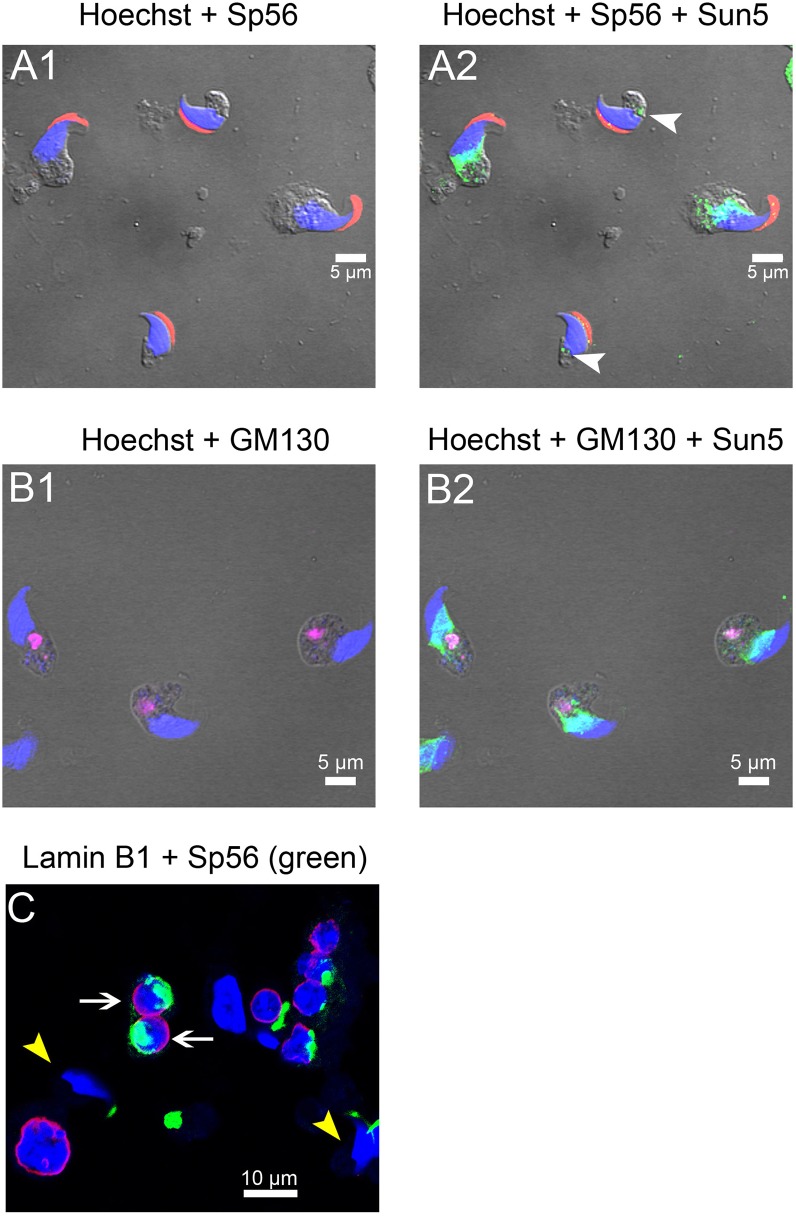
Sun5 is removed during spermiogenesis along an antero-caudal axis. **(A)** In early condensing spermatids, Sun5 is located at the base of the nucleus. Elongating spermatids co-stained with anti-Sp56 antibodies (A1, red) and anti-Sun5 Ab1 (A2, green) and counterstained with Hoechst to evidence the nucleus (blue). Arrow heads show specific Sun5 staining at the implantation fossa in late condensing spermatids. **(B)** In early condensing spermatids, Sun5 is no longer present in Golgi apparatus. Elongating spermatids co-stained with anti-GM130 antibodies (B1, purple) and anti-Sun5 Ab1 (B2, green) and counterstained with Hoechst to evidence the nucleus (blue). **(C)** In round spermatids (white arrows), Lamin B1 (purple) is not located in front of the acrosomal vesicle (evidenced by anti-Sp56 antibodies green), showing that lamin B1 and Sun5 were both excluded from the acrosomal area. In early condensing spermatids (yellow arrow heads), Lamin B1 (purple) is absent. Cells were counterstained with Hoechst to evidence the nucleus (blue).

During spermiogenesis, and particularly during spermatid elongation, Sun5 was moved back along an antero-posterior axis (Fig. 5AB). This movement seems to occur earlier at the border of the acrosomal cap than on the opposite side of the spermatid. Again, no Sun5 staining was observed in front of the acrosome (Fig. 5AB). At this stage, Sun5 was no longer co-localized with the Cis-Golgi, suggesting that most Sun5 proteins had joined their final target in the nuclear envelope (Fig. 5B). It was reported that Sun1 protein interacts with lamin A [15] and interestingly we show that Sun5 presented a similar localization to lamin B1 in the round spermatids, that is, close to the NE, except in front of the acrosomal vesicle (Fig. 5C, white arrows). This may suggest that Sun5 is a potential partner of lamin B1. Lamin B1 is however quickly removed at the end of the round spermatid stage and no fluorescent signal was observed in early condensing spermatids (Fig. 5C, yellow arrow heads). This result thus demonstrates that Sun5 movement during late spermiogenesis is not dependent of lamin B1.

**Fig 6:**
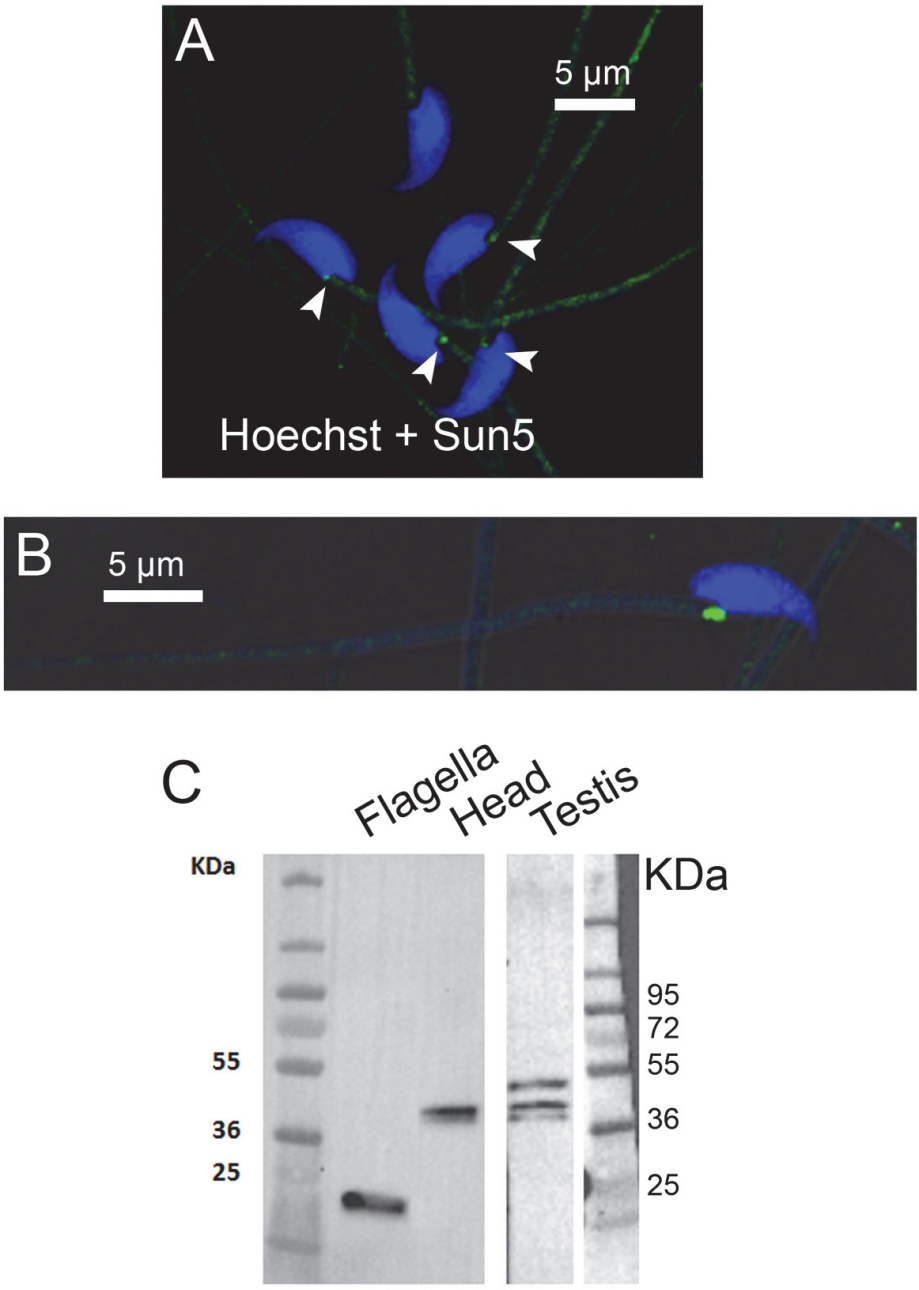
Epididymal sperm presents a punctiform staining at the implantation fossa. **(A, B)** Sun5 staining is located at the implantation fossa in epididymal sperm (arrow heads). **(C)** Sperm were fractionated by mild sonication allowing separating head and flagella fractions and resolved proteins by SDS PAGE of both fractions were subjected to Western blotting analysis with Ab2 Sun5 antibody. As control, testis nuclear protein extracts were subjected to Western blotting analysis in the same trial.

Finally, we investigated the presence of Sun5 in mature epididymal sperm. Due to the great compaction of the sperm nucleus and the structures of the peri-nuclear theca, the staining of the NE may be difficult. Sperm nuclei were thus decondensed with 10 mM DTT. We have previously shown that this method allows evidencing protamine, a protein deeply embedded in the nucleus [16]. In the epididymal sperm, Sun5 was observed in front of the sperm implantation fossa (Fig. 6AB, white arrow heads). This specific pattern of localization was already observable in late condensing spermatids (Fig. 5A2, white arrow heads). It is worth noting that a similar staining was observed on sperm not treated with DTT, and thus does not correspond to an artifact induced by DTT treatment. A faint staining was also observed in the flagellum of mature epididymal sperm (Fig. 6AB). In order to validate both staining, we compared the band patterns obtained by Western blotting of heads and flagella fractions (Fig. 6C). The heads and flagella fractions were obtained by mild sonication. In the flagella fraction, the Sun5 antibodies immunodecorated a band at 25 kDa only, suggesting that flagellum staining was not specific because Sun5 MW is around 40 kDa.

**Fig 7:**
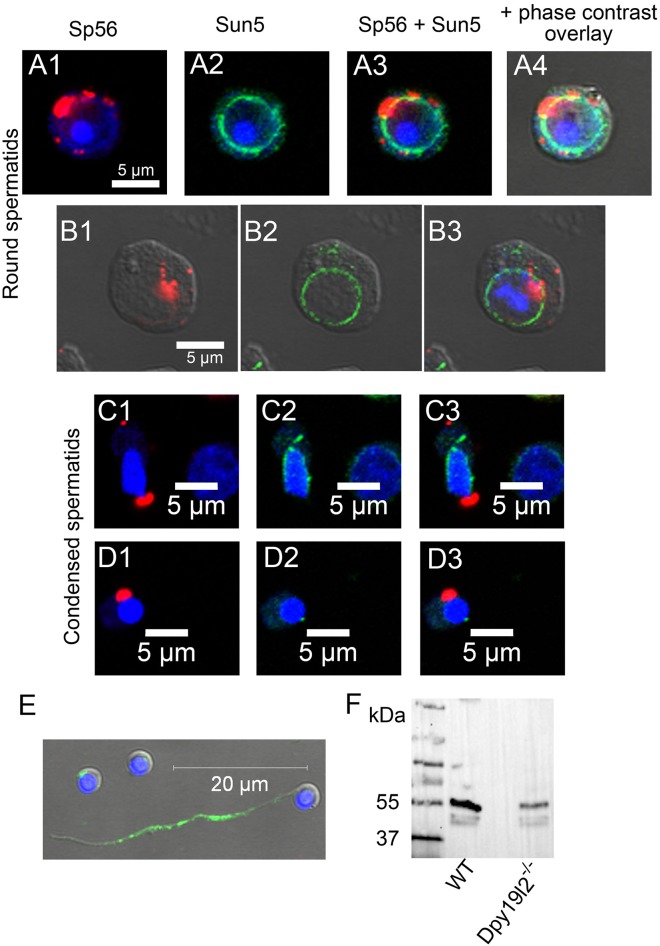
Detachment of the acrosome in spermatids from *Dpy19l2* KO males leads to a relocalization of Sun5. **(A, B)** Round spermatids co-stained with anti-Sp56 antibodies (A1, B1, red) and anti-Sun5 Ab1 (A2, B2, green) and counterstained with Hoechst to evidence the nucleus (blue). A3 corresponds to overlay and A4 and B3 correspond to overlay + phase contrast. **(C)** Sun5 removal during spermiogenesis along the antero-caudal axis is not modified in *Dpy19l2* KO condensed spermatids. Elongating *Dpy19l2* KO spermatids co-stained with anti-Sp56 antibody (C1, red) and anti-Sun5 Ab1 (C2, green) and counterstained with Hoechst to evidence the nucleus (blue); C3 overlay. **(D)** Similar stainings in fully condensed spermatids showing the almost complete vanishing of Sun5. **(E)** Absence of Sun5 staining in *Dpy19l2* KO sperm head. **(F)** Western blot of nuclear protein extracts from WT and *Dpy19l2* KO testis revealed with anti-Sun5 Ab1. The absence of Dpy19l2 did not modify Sun5 glycosylation pattern.

All the data presented above show that Sun5 is produced early during the meiosis and traffics through the Golgi apparatus before reaching the NE of the round spermatid. The localization of Sun5 is highly controlled since it is excluded from the NE facing the acrosome. To get better insights in the mechanism allowing its exclusion from this specific area, we measured Sun5 localization in spermatogenic cells from *Dpy19l2* KO males; Dpy19l2 is a transmembrane protein of the INM and its absence leads to acrosome detachment and reorganization of proteins of the NE [5]. The absence of Dpy19l2 led to a striking relocalization of Sun5 towards the nuclear envelope area previously involved in acrosomal vesicle attachment, as witnessed by the full ring shape of Sun5 staining around the nucleus (Fig. 7AB). This relocalization, which is concomitant with acrosome vesicle detachment, confirms that Sun5 is excluded from the acrosomal vesicle attachment area. At later stages, the absence of Dpy19l2 did not modify the movement of Sun5 along the antero-posterior axis (Fig. 7C) and the vanishing of the protein in late condensing spermatids (Fig. 7D). In epididymal sperm from *Dpy19l2* KO males, the specific staining in the implantation fossa was lost in most of sperm and the flagellum staining was similar to that observed in WT flagellum (Fig. 7E) and is thus likely to be unspecific as well. Finally, a recent report showed that *Dpy19*, the ortholog of *Dpy19l2* in C. elegans, is a C-mannosyltransferase [17]. Sun5, as a nuclear envelope protein may be glycosylated by Dpy19l2. To test this hypothesis, we observed the pattern of Sun5 bands in WB of a nuclear extract from Dpy19l2 KO testis. The absence of Dpy19l2 did not modify the pattern of post-translational modifications (Fig. 7F), both the doublet at 40 KDa and the band at 55 KDa were observed. This result suggests that Dpy19l2 is not involved in the glycolysation process of Sun5.

## Discussion

During spermiogenesis, the spermatid nucleus is deeply modified. The intervening modifications involve changes in DNA packaging, nuclear shape and a reorganization of the NE structure. However, the function of the integral membrane proteins of the NE and in particular of Sun proteins during spermatid elongation remains poorly understood and deserve to be investigated. As indicated in the introduction, all Sun proteins are present in spermatogenic cells and their expression and localization are strongly controlled. However, their distinct roles in spermiogenesis remain to be unraveled [9,18].

Herein, we focused our work on Sun5 localization during spermatogenesis. We showed that Sun5 shares common properties with other Sun proteins. First, as described for Sun1 and Sun3, Sun5 is located in the NE of the round spermatids. Moreover, we demonstrated that like the other Sun proteins studied in spermatogenic cells so far [9], Sun5 is excluded from the region facing the acrosomal vesicle. Sun proteins thus share common structural properties allowing their positioning into the NE and their exclusion from the acrosomal vesicle area. The molecular determinants of Sun exclusion from the acrosomal vesicle area have not yet been characterized. Furthermore, we showed that the detachment of the acrosomal vesicle due to the absence of Dpy19l2 led to an important relocalization of Sun5. In a previous report we showed that acrosome detachment leads to the vanishing of a specific nucleoplasmic structure called the nuclear dense lamina which does not contain any lamin [5]. This structure could be involved in Sun exclusion and would deserve further studies. Sun3 and Sun1 present a complementary localization in round spermatids: Sun1 is located at the posterior pole and Sun3 at an intermediate location between the anterior and the posterior poles [9]. The molecular cause of such mutual exclusion is not understood so far. In comparison, we showed that the area of Sun5 localization is unique and encompasses Sun1 and Sun3 localizations. Moreover, Sun5 presents some specific features in comparison to other Sun proteins. First, epididymal sperm present a staining in the implantation fossa, a localization where Sun1 is absent [9]. As a nuclear envelope protein, the location of Sun5 in this peculiar area is coherent with the presence of a redundant nuclear envelope at the base of the sperm head as showed in bull and mouse [19]. Although epididymal sperm exhibit compact structures, it is worth noting that Sun5 staining in sperm were performed in conditions allowing us to evidence other proteins (like protamines) located deeper in the nucleus [16], and the presence of Sun5 in other locations is unlikely. Concerning Sun5 staining in the flagellum of mature epididymal sperm, the anti-Sun5 Ab1 antibodies immunodecorated the flagellum in both WT and *Dpy19l2* KO mature sperm. However, we observed no bands at 40 or 55 KDa in Western blot carried out with the flagella fraction. On the other hand a band at 25 KDa was observed. This band is likely to be non-specific because the smallest alternative transcript identified (XP_006500438), which code for proteins with a molecular weight of 29 KDa does not contains the TLPEDTTHSGRPRRGVQRSY sequence found on the main transcript and which was used for antibody design. It is important to notice that this 25 KDa band was not observed in Western blots performed from whole testis proteins because there are relatively few mature sperms in the testes compared to other cell types and the blots were carried out on enriched nuclear proteins.

In this report, we also showed that Sun5 is clearly present in the cytoplasm at the pachytene stage in an uncharacterized membranous compartment. It is worth noting that at this stage, Western blotting experiment evidenced only one band around 40 KDa, a result coherent with its transit through the Golgi apparatus at the round spermatid stage. This uncharacterized membranous compartment may correspond to the endoplasmic reticulum (ER)-Golgi intermediate compartment (ERGIC) because it has been shown that integral proteins of the NE including emerin, another protein of the INM, transit through the ERGIC before retrograde transport to the nuclear envelope [20]. Such a location was already suggested for Sun1 [21]. The transit of Sun5 through the Golgi is associated with deep post-translational modifications. In HEK cells and spermatogenic cells, three different antibodies immunodecorated two close bands around 40 kDa. Two of these antibodies target different parts of the mouse Sun5 protein and one the DDK-tag of the human Sun5-DDK protein. It is thus very unlikely that these bands correspond to a non-specific staining, but rather correspond to post-translational modifications. Interestingly, Sun2 is heavily phosphorylated in HeLa cells treated with phosphatase inhibitors [22]. Moreover, in spermatogenic cells, a new band at 55 kDa was immunodecorated. Again this band was revealed by two different primary antibodies revealed by two different secondary HRP antibodies (rabbit versus rat) and unlikely correspond to an unspecific marking. Moreover, the expression of Sun5 has been extensively studied and 5 splice variants have been identified. The main transcript (NM_029599.1) codes for a 348 amino acids (aa) protein (NP_083875.1) with an estimated molecular weight of 39 805 Da. Four alternative transcripts have been identified which code for proteins ranging from 259 to 382 aa with molecular weight ranging from 29545 to 43069 Da (XP_006500438, XP_006500436.1, XP_006500437.1, XP_006500435.1). It is extremely unlikely that a larger transcript exists. Finally this band was no longer observable after a treatment with deglycolysation enzymes. Altogether these data show that Sun5 is post-translationally modified. To our knowledge, it is the first description of glycosyl modifications of a Sun protein. The fact that Sun5 is post-translationally modified is confirmed by the fact that numerous putative glycosylation sites are predicted using programs detecting post-translational modifications (http://www.expasy.org/proteomics/post-translational_modification). Although the cellular function associated with this glycosylation is currently unknown, it is important to notice that glycosylation of nuclear envelope membrane proteins including nucleoporins is important for the functional integrity of the nuclear envelope [23]. Interestingly, antibodies also evidenced a band around 95 KDa, which was no longer observable when reducing conditions were increased. This band likely corresponds to a complex containing Sun5. Because the MW of the complex is close to the MW of the Sun 5 dimer and because it has clearly been shown that Sun proteins are associated in oligomers [24], this band likely corresponds to a Sun5 dimer. Remnants of dimers or trimers in WB is very usual for oligomers in weak reducing conditions (without boiling), especially when proteins are difficult to solubilize (it is the case for Sun5, as showed previously [11]). Such a band was also observed in WB performed with extracts from HEK cells transfected with Sun5-DDK plasmid and revealed with a third primary antibody (anti-DDK) and a different secondary antibody (anti mouse HRP). Because Sun proteins have been described to mainly function as trimers [22], the observed dimers may therefore correspond to a partial Sun5 trimer dissociation during protein solubilization. This suggests that Sun5, like other Sun proteins, might also be associated in trimers. The fact that no trimers were observed could be due to a low solubility of the trimeric structure.

The results presented above are clearly different from what was described in previous reports showing that Sun5 is mainly expressed during meiosis in the reticulum [12] and that Sun5 is located in the NE facing the acrosome and is not post-translationnally modified [11]. The main difference between our experiments and previous reports was the use of different antibodies. We did test the commercially available antibody used in the previous reports [11] and, despite numerous trials with different conditions, we were unable to reproduce the previously published data. The results presented herein have been obtained using three different antibodies, one of which was new. Their specificity was challenged using two different plasmids containing either mouse Sun5 or human SUN5 DNA sequences in both IHC and Western blot experiments. In IHC, a coherent staining was observed as a ring surrounding the nucleus. A similar staining had already been described with an anti DDK antibody when cells were transfected with Sun5-DDK [5]. We never observed any staining in non transfected cells. The results obtained with different antibodies combinations were highly concordant and altogether, these control experiments brought a high level of confidence in our data.

So far, the interactions between the organelles and the nucleus have always been dependent on the formation of LINC complexes due to the binding of Sun and Kash domain proteins. Herein, we demonstrated by IHC that Sun5 is undetectable in the NE facing the acrosome and thus that the attachment of the acroplaxome on the nucleus does not likely depend on the formation of a LINC complexes involving Sun5. Moreover, several publications on the localization of other Sun proteins in spermatogenic cells, shows that they are also absent from the NE facing the acrosome [8,9,18]. Altogether, these results suggest that the attachment of the acroplaxome on the NE does not likely depend on Sun proteins and seems thus unique. In conclusion, the only protein described so far to be involved in acrosomal attachment remains Dpy19l2. The molecular partners of Dpy19l2 located in the nucleoplasm and in the ONM remain to be characterized. Finally, this data will avoid inverse genetic studies focusing on this protein in the framework of globozoospermia.

## Material and Methods

### Ethics Statement

All animal procedures were run according to the French guidelines on the use of animals in scientific investigations with the approval of the local Ethical Committee, (Grenoble-Institut des Neurosciences—ethical committee, Study agreement number 004). Mice were killed by cervical dislocation.

### Animals

*Dpy19l2* ^*−/−*^ mice were obtained from Mutant Mouse Regional Resource Center, University of California, Davis, CA. Although gene knock-out had been checked by PCR/Southern (http://mmrrc.mousebiology.org/doc/di_032274PCR_Protocol.pdf), we confirmed by RT-PCR that Dpy19l2 transcripts were absent [5].

### Cell Culture and transfection

HEK-293 and NIH/3T3 cells were grown in Dulbecco’s Modified Eagle’s Medium supplemented with 10% FBS (Invitrogen, France) and transiently transfected either with human orthologs Cter-DDK-tagged Sun5 or mouse Sun5 containing pCMV6 plasmids (from Origene, Rockville, MD, US), using Lipofectamine 2000 Transfection Reagent (Invitrogen) according to the manufacturer’s instruction. Two days after transfection, transfected cells were fixed with 4% paraformaldehyde (PFA) before immunochemistry experiments.

### Testicular Cell dissociation

C57BL6 male or *Dpy19l2* KO mice (8 weeks old) were killed by cervical dislocation. The testes were surgically removed and placed in Dulbecco's phosphate-buffered saline (PBS) at room temperature. The tunica albunigea was removed from the testes with sterile forceps and discarded. Then, the testes were incubated in 3 ml of a solution containing 1 mg/ml collagenase and 2 mM CaCl_2_, 12.1 mM Glucose, 10 mM HEPES, 5 mM KCl, 1 mM MgCl_2_, 6 mM Na-Lactate, 150 mM NaCl, 1 mM NaH_2_PO_4_, 12 mM NaHCO_3_ pH = 7) and agitated horizontally at a maximum of 120 rpm for 30 min at 25°C. The dispersed seminiferous tubules were then washed with PBS and cut thinly. Cells were dissociated by gently pipetting, filtered through a 70 μm filter and then pelleted by centrifugation at 500 g for 10 minutes. Cells were suspended in 1 ml PBS and fixed with 4% PFA solution, washed with PBS and finally layered onto polylysine-coated slides. For WB experiments of purified pachytene spermatocytes, cell suspensions were loaded into a sedimentation chamber according to procedures developed for murine spermatogenic cells for velocity sedimentation under unit gravity separation [25].

### Nuclear protein preparation

Enriched nuclear proteins samples were prepared essentially as described by Korfali et al [26], using dissociated testicular cells. In brief, cells were dispersed in 3 ml of ice-cold hypotonic buffer (10 mM HEPES pH 7.4, 1.5 mM MgCl_2_, 10 mM KCl, 2 mM DTT) containing Complete protease inhibitor cocktail (Roche) on ice. After 10 min, cells were lysed by application of 20 strokes in a Dounce hand homogenizer. Nuclei were then separated from the cells by a sucrose gradient (0.22 M to 0.9 M). Nuclei were washed in PBS buffer and pelleted at 2000 g at 4°C for 15 min. Nuclei were then solubilized in buffer containing 5 mM MgCl_2_, 1% triton X-100, 100 U/ml DNAse and complete protease inhibitor cocktail (Roche) incubated for 4 hours at 4°C under agitation. After centrifugation at 20 000 g for 30 min at 4°C, the soluble supernatant was conserved and subjected to SDS-PAGE.

### Western Blotting analysis

Proteins of enriched nuclear preparation were diluted in ultra pure water and mixed with 5X protein sample buffer (62 mM Tris-HCl pH 6.8, 2% SDS, 10% glycerol, 5% ß-mercaptoethanol, and 0.05% bromophenol blue as the tracking dye), and samples were heated at 95°C for 5 minutes when required. Samples were then separated on 4–20% SDS-PAGE gels and transferred into PVDF membranes (Millipore) using a Mini Trans-Blot Cell (Bio-Rad). The membranes were blocked in 6% non-fat dry milk in PBS 0.1% Tween and incubated overnight at 4°C with Ab1 anti-Sun5 (1/1000), Ab2 anti-Sun5 (1:500) or anti-DDK (1:1000) antibodies; this was followed by 30 min of incubation with a matched horseradish peroxidase labeled secondary antibody (1/10,000). Immunoreactivity was detected using chemiluminescence detection kit reagents and a Chimidoc Station (Biorad). Western blotting procedures were repeated at least 3 times per sample.

### Immunohistochemistry (IHC)

For IHC, heat antigen retrieval was performed by boiling slides immersed in 0.01 M sodium citrate buffer, 0.05% Tween 20, pH 6.0 during ∼15 min. Incubation with primary antibodies was performed overnight at 4°C in PBS with 2% goat serum, 1% bovine serum albumin, 0.1% Triton X-100. Slides were then washed and incubated with secondary antibodies for 1 hour at room temperature and counterstained with Hoechst 33342. Images were taken by confocal microscopy (Zeiss LSM 710), and processed using Zen 2009.

### Antibodies

Mouse Sperm Protein Sp56 Monoclonal Antibodies and Golgi matrix protein GM130 (610822), two monoclonal antibodies raised in mouse, were from QED Bioscience; Lamin B1 antibodies were from Abcam (Ab16048); DDK-tag antibodies were from Origene, anti-KDEL from Calbiochem Millipore (Mouse mAb 10C3, 1/200). Two Sun5 antibodies were used: Ab1 corresponds to Spag4l antibody raised in rabbit from Proteintech antibodies (17495–1-AP) and was used from 1/100 to 1/200 in IHC and Ab2 corresponds to laboratory designed antibodies targeting the peptide CTLPEDTTHSGRPRRGVQRSY and corresponding to amino acids 11–31 of the mouse Sun5 and raised in rat.

### Enzymatic deglycosylation

Samples (50 μg of total protein per reaction) were incubated at 37°C for 15 hours under non reducing conditions with a mixture of different glycosidases (PNGase F, Neuraminidase, O-glycosidase, ß 1–4 Galactosidase, ß- N-Acetylglucosaminidase (Protein Deglycosylation Kit, Promega) and protease inhibitors (Roche). Deglycosylated proteins were separated on 4–20% gradient SDS-polyacrylamide gel (Bio-Rad) followed by western blot analysis, using the same antisera described above for Sun 5 protein. The control sample was incubated without glycosidase mix and a positively glycosylated protein (fetuin) was provided as a substrate control.

### Fractionation of sperm heads and tails

Cauda epididymal sperm, obtained from male mice (OF1) was collected in PBS buffer and allowed to swim for 10 minutes at 37°C. Sperm heads and tails were separated by mild sonication on ice (five times ON, five times OFF) using Ultrasonic sonicator (Delta labo).The sample was then layered on a discontinuous Percoll density gradient composed of six layers containing 100, 60, 34, 26, 23, and 21% Percoll. All of the percoll solutions were diluted in M2 medium (Sigma-Aldrich, France). The gradient was centrifuged at 600 g for 20 min at 4°C. After centrifugation, sperm heads were recovered in the fraction 100% whereas sperm tails were recovered in the fraction 60%. Both samples were washed in PBS buffer, and then the pellets were resuspended in Laemmli sample buffer without ß-mercaptoethanol, and boiled for 5 minutes at 100°C. After centrifugation, 5% ß-mercaptoethanol was added to the supernatants, and the mixture was boiled again for 5 minutes. Protein extracts were loaded and subjected to SDS-PAGE as described above.

### Funding

This study was supported by grants from Gravit Foundation (to CA), from the Agence National de la Recherche (Grant ICG2I to PR and CA).

## Supporting Information

S1 Fig No staining is observed in spermatogenic cells treated with only secondary antibody. No staining is observed in pachytene spermatocytes (PS), round spermatids (RS) or elongating spermatids (SE).
(JPG)

S2 Fig Co-staining of pachytene spermatocytes with KDEL antibodies (reticulum marker, red) and Sun 5 antibodies (green). **(A)** In pachytene, a punctiform staining is observed within the cytoplasm but no staining were observed around the nucleus (A1). The reticulum staining is observed within the reticulum (A2). (A3) overlay. Most of the green and red staining were not superposed. Nuclei were counterstained with Hoechst (blue). **(B)** Similar pattern of KDEL and Sun5 staining in a different pachytene spermatocyte.
(JPG)

S3 Fig Sun5 is located in the nuclear envelope in round spermatids and excluded from the NE facing the acrosome (A-E). Round spermatids co-stained with anti-Sp56 (red) and anti-Sun5 Ab1 (green) and counterstained with Hoechst to evidence the nucleus (blue). **A-D** panels show examples of the absence of staining in the NE facing the acrosome. **A** and **E** panels: examples of cells showing that Sun5 staining is located more externally than Sp56 Staining. Arrow heads indicate the Sun5 staining which was not bound to the nuclear envelope.
(JPG)

S4 Fig NE-unbound Sun5 staining co-localizes with the Golgi apparatus in round spermatids. Round spermatids were co-stained with anti-Sun5 antibody (**A1**, **B1**, **C1**, green staining) and with anti-GM130 antibody to evidence the Cis-Golgi (**A2**, **B2, C2**, red staining) and counterstained with Hoechst to evidence the nucleus. Overlays of GM130 and Sun 5 staining show that NE-unbound Sun5 staining co-localizes with the Golgi Apparatus (**A3**, **B3**, **C3**, arrow heads).
(JPG)

S5 Fig Control data of deglycosylation experiments. **(A)** Control showing that our deglycosylation protocol is able to remove glycosyl residues of bovine fetuin. (−) corresponds to fetuin incubated at 37°C for 15 hours in the absence of enzymes and (+) to fetuin incubated at 37°C for 15 hours with a mixture containing different glycosidases. **(B)** Protein loads were controlled with TGX stain free precast gels. Lane 1 corresponds to testis proteins which were conserved at 4°C during the deglycosylation protocol, lane 2 to testis proteins incubated at 37°C for 15 hours with a mixture of different glycosidases, lane 3 to testis proteins incubated at 37°C for 15 hours in the buffer without enzymes and lane 4 to molecular weights. **(C)** Protein load, corresponding to the Western blot presented in Fig. 4D, demonstrates that similar amounts of protein were loaded.
(JPG)
