# Correction: Dynamics of Sun5 Localization during Spermatogenesis in Wild Type and *Dpy19l2* Knock-Out Mice Indicates That Sun5 Is Not Involved in Acrosome Attachment to the Nuclear Envelope

**DOI:** 10.1371/journal.pone.0125452

**Published:** 2015-04-10

**Authors:** The PLOS ONE Staff

There is an error in the third author’s name. The correct surname for the third author is: Abi Nahed. The correct given name is: Roland. The correct citation is: Yassine S, Escoffier J, Abi Nahed R, Pierre V, Karaouzene T, Ray PF, et al. (2015) Dynamics of Sun5 Localization during Spermatogenesis in Wild Type and *Dpy19l2* Knock-Out Mice Indicates That Sun5 Is Not Involved in Acrosome Attachment to the Nuclear Envelope. PLoS ONE 10(3): e0118698. doi:10.1371/journal.pone.0118698

The publisher apologizes for the error.
