# Supplementary figures and images for: Dynamics of Sun5 Localization during Spermatogenesis in Wild Type and Dpy19l2 Knock-Out Mice Indicates That Sun5 Is Not Involved in Acrosome Attachment to the Nuclear Envelope
Source: PLoS One. 2015 Mar 16;10(3):e0118698. doi: 10.1371/journal.pone.0118698 (PMC4361733; doi:10.1371/journal.pone.0118698)

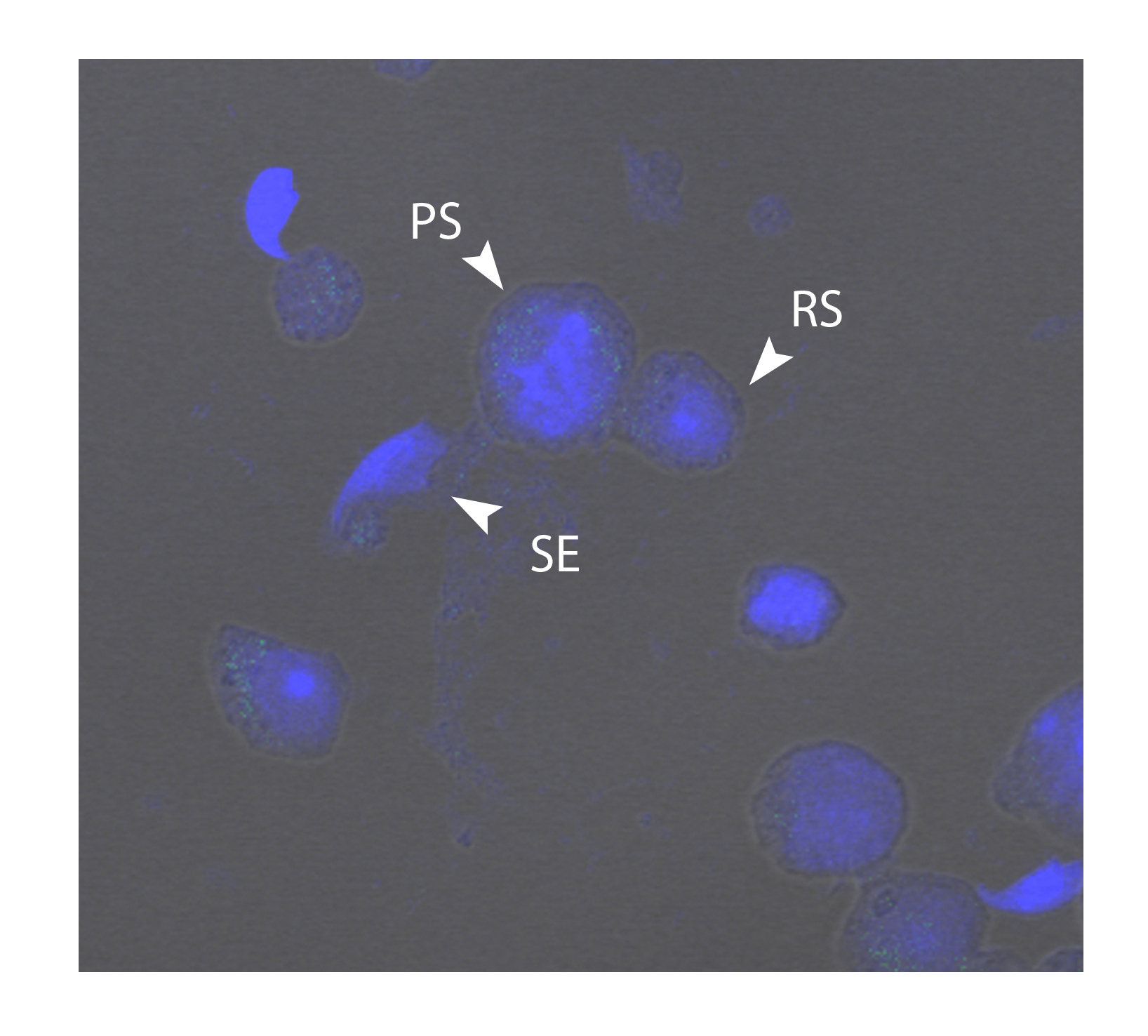

Supplement: S1 Fig — No staining is observed in pachytene spermatocytes (PS), round spermatids (RS) or elongating spermatids (SE). (JPG) [file pone.0118698.s001.jpg]

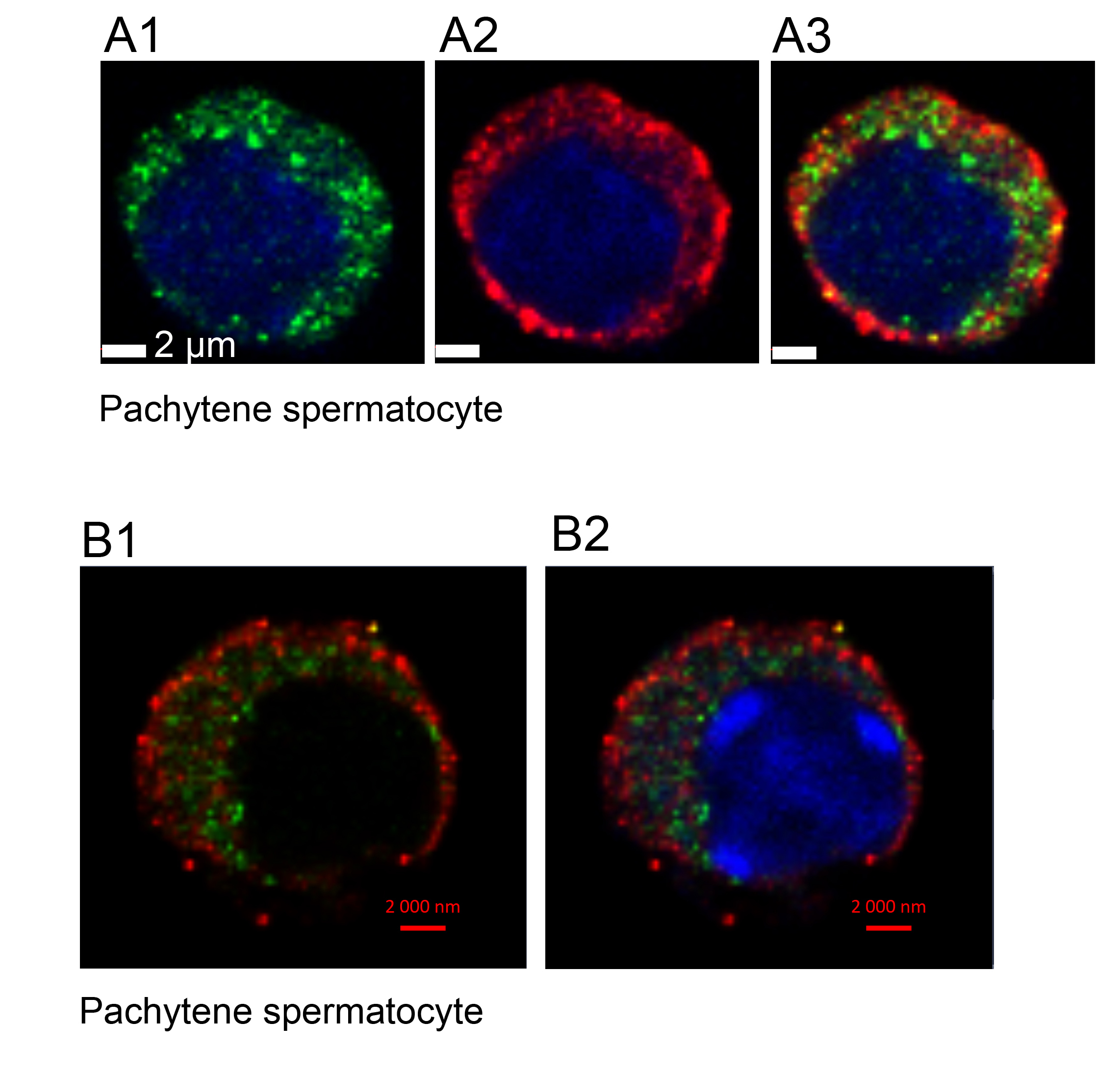

Supplement: S2 Fig — (A) In pachytene, a punctiform staining is observed within the cytoplasm but no staining were observed around the nucleus (A1). The reticulum staining is observed within the reticulum (A2). (A3) overlay. Most of the green and red staining were not superposed. Nuclei were counterstained with Hoechst (blue). (B) Similar pattern of KDEL and Sun5 staining in a different pachytene spermatocyte. (JPG) [file pone.0118698.s002.jpg]

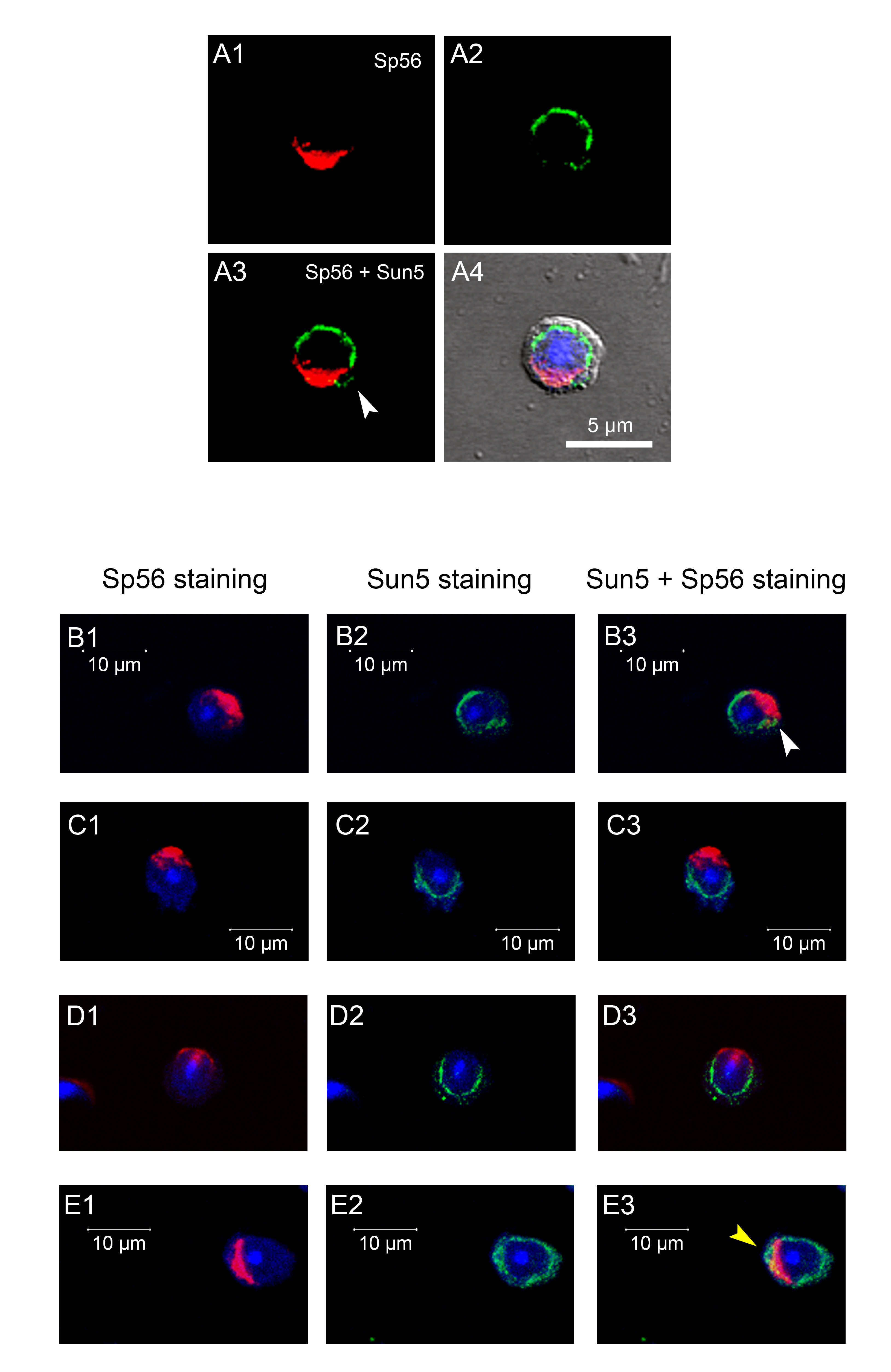

Supplement: S3 Fig — Round spermatids co-stained with anti-Sp56 (red) and anti-Sun5 Ab1 (green) and counterstained with Hoechst to evidence the nucleus (blue). A-D panels show examples of the absence of staining in the NE facing the acrosome. A and E panels: examples of cells showing that Sun5 staining is located more externally than Sp56 Staining. Arrow heads indicate the Sun5 staining which was not bound to the nuclear envelope. (JPG) [file pone.0118698.s003.jpg]

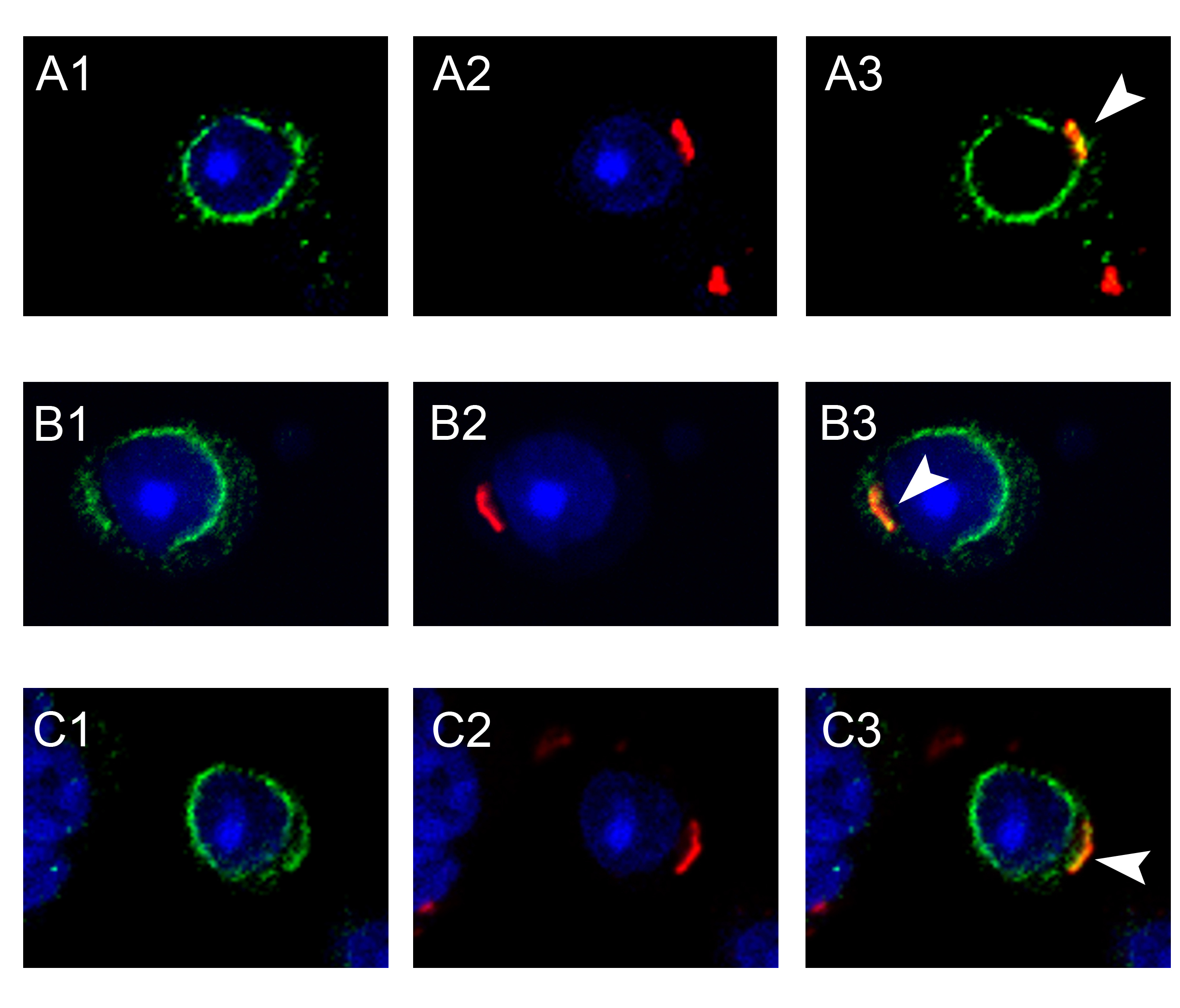

Supplement: S4 Fig — Round spermatids were co-stained with anti-Sun5 antibody (A1, B1, C1, green staining) and with anti-GM130 antibody to evidence the Cis-Golgi (A2, B2, C2, red staining) and counterstained with Hoechst to evidence the nucleus. Overlays of GM130 and Sun 5 staining show that NE-unbound Sun5 staining co-localizes with the Golgi Apparatus (A3, B3, C3, arrow heads). (JPG) [file pone.0118698.s004.jpg]

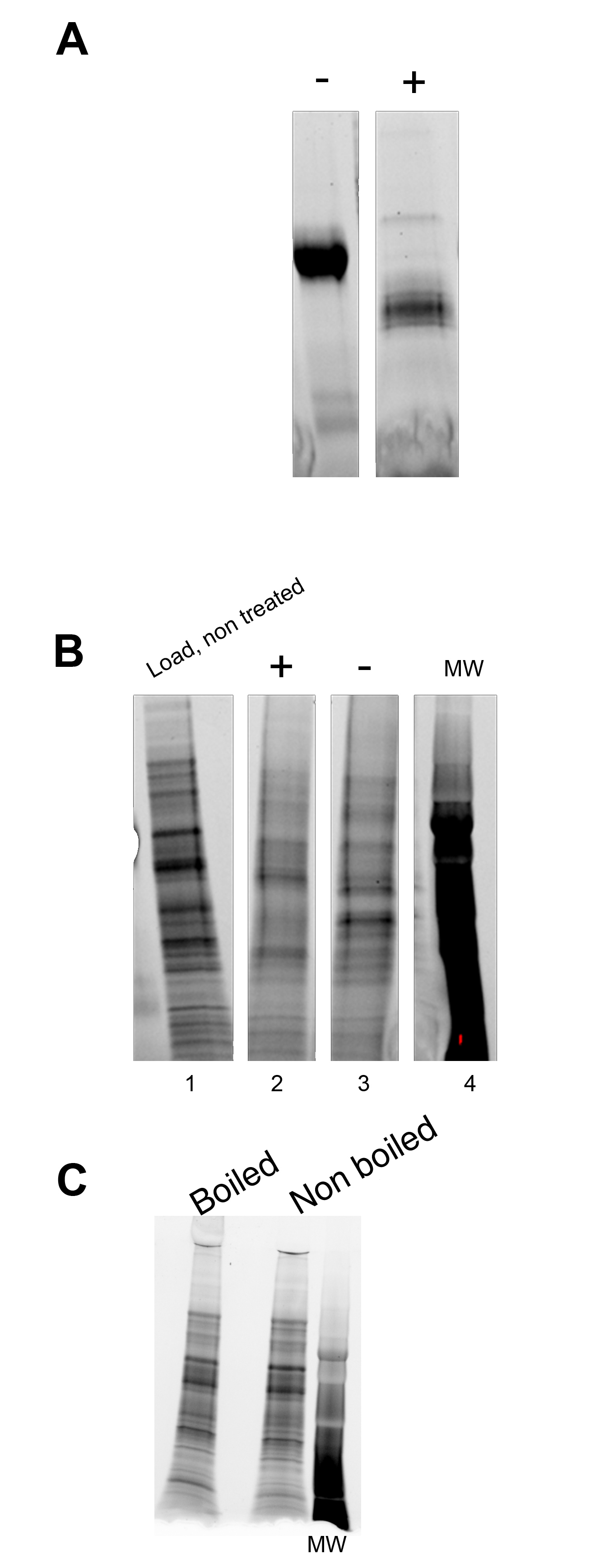

Supplement: S5 Fig — (A) Control showing that our deglycosylation protocol is able to remove glycosyl residues of bovine fetuin. (−) corresponds to fetuin incubated at 37°C for 15 hours in the absence of enzymes and (+) to fetuin incubated at 37°C for 15 hours with a mixture containing different glycosidases. (B) Protein loads were controlled with TGX stain free precast gels. Lane 1 corresponds to testis proteins which were conserved at 4°C during the deglycosylation protocol, lane 2 to testis proteins incubated at 37°C for 15 hours with a mixture of different glycosidases, lane 3 to testis proteins incubated at 37°C for 15 hours in the buffer without enzymes and lane 4 to molecular weights. (C) Protein load, corresponding to the Western blot presented in Fig. 4D, demonstrates that similar amounts of protein were loaded. (JPG) [file pone.0118698.s005.jpg]
